# Hip Flexor Muscle Activation During Common Rehabilitation and Strength Exercises

**DOI:** 10.3390/jcm13216617

**Published:** 2024-11-04

**Authors:** Jessica Juan, Gretchen Leff, Kate Kevorken, Michael Jeanfavre

**Affiliations:** Stanford Health Care, Outpatient Orthopedic & Sports Medicine Rehabilitation Department, Redwood City, CA 94063, USA; kkevorken@stanfordhealthcare.org

**Keywords:** iliopsoas, electromyography, exercise therapy, biofeedback, hip flexor

## Abstract

**Background/Objectives:** The iliopsoas muscle plays an essential role in lumbopelvic and hip anterior stability, which is particularly important in the presence of limited osseous acetabular coverage anteriorly as in hip dysplasia and/or hip micro-instability. The purpose of this systematic review is to (1) describe iliopsoas activation levels during common rehabilitation exercises and (2) provide an evidence-based exercise progression for strengthening the iliopsoas based on electromyography (EMG) studies. **Methods:** In total, 109 healthy adult participants ranging from ages 20 to 40 were included in nine studies. PubMed, CINAHL, and Embase databases were systematically searched for EMG studies of the psoas, iliacus, or combined iliopsoas during specific exercise. The Modified Downs and Black Checklist was used to perform a risk of bias assessment. PROSPERO guidelines were followed. **Results:** Nine studies were included. Findings suggest that the iliopsoas is increasingly activated in ranges of hip flexion of 30–60°, particularly with leg lowering/raising exercises. Briefly, >60% MVIC activity of the iliopsoas was reported in the active straight leg raise (ASLR) in ranges around 60° of hip flexion, as well as with supine hip flexion and leg lifts. In total, 40–60% MVIC was found in exercises including the mid-range of the ASLR around 45° of hip flexion and lifting a straight trunk while in a hip flexed position. **Conclusions:** The findings suggest that exercises in increased hip flexion provide greater activation of the iliopsoas compared to exercises where the trunk is moving on the lower extremity. Iliopsoas activation can be incrementally progressed from closed to open kinetic chain exercises, and eventually to the addition of external loads. The proposed exercise program interprets the results and offers immediate translation into clinical practice.

## 1. Introduction

Research labs around the world describe the anatomical and physiological importance of the iliacus muscle, comprising the iliacus, psoas major, and psoas minor [1,2]. With the iliacus, psoas major, and psoas minor, the iliopsoas serves as a primary hip flexor, and contributes to hip external rotation as well as trunk lateral flexion [1,2]. It is considered a core muscle due to its attachments, and functions to stabilize the trunk as well as the pelvis [2]. The psoas major has proximal attachments of the transverse processes, intervertebral disks, and vertebral bodies of T5-L5, while the iliacus has proximal attachments from the superior two thirds of the iliac fossa, sacral ala, and ventral lip of the iliac crest [1]. These two muscles combine to become the iliopsoas at levels L5-S2 and then insert onto the lesser trochanter of the femur, creating the iliopsoas tendon [1]. The psoas minor has a proximal attachment at the T12-L1 vertebrae and a distal attachment of the iliopubic eminence [1]. The iliopsoas functions as both a trunk and hip stabilizer due to its anatomical position, where it is in close proximity to the anterior labrum of the hip joint. In addition to functioning as a hip flexor, a study out of Melbourne Australia described that the iliopsoas also acts as an important anterior joint stabilizer by applying posterior compressive forces to the femoral head, reducing extraneous shear force and lowering the risk of labral or capsular injury in cases of micro-instability. They explain that this stabilization can be likened to the role of the rotator cuff in the shoulder [3]. The present systematic review aims to delineate iliopsoas activation levels during common rehabilitation exercises and offer an evidence-based exercise progression for strengthening the iliopsoas based on EMG studies.

Conditions like adult hip dysplasia and hip micro-instability increase reliance on anterior dynamic stabilizers such as the iliopsoas. Adult hip dysplasia, affecting around 5.2% of the population, is characterized by insufficient coverage of the femoral head by the acetabulum [4]. Conversely, hip micro-instability involves increased femoral head movement within the acetabulum, potentially due to joint laxity [5]. While the prevalence of hip micro-instability remains undefined due to challenges in identifying asymptomatic cases, joint hypermobility is considered one contributing factor with an incidence of 18.9% [6]. Moreover, the presence of injury or tears of the acetabular labrum, which provides native joint stability by creating a vacuum seal between the femoral head and acetabulum, can also lead to hypermobility of the hip joint [7]. Evidence indicates that in both conditions, the iliopsoas plays a crucial role in compensating for decreased anterior joint stability, mitigating the risk of injury to surrounding soft tissue structures. In hip dysplasia, the hypertrophy of the iliocapsularis, a muscle that is clinically and radiographically indistinguishable from the iliopsoas, suggests increased utilization of the iliopsoas as an anterior stabilizer compared to healthy individuals [8].

Two other types of populations in which iliopsoas function may be pertinent are populations with a presence of total hip arthroplasty (THA), and more athletic populations during running speeds (i.e., particularly during running speeds exceeding >7 m/s when increasing cadence and stride frequency) [9,10]. Iliopsoas tendinopathy is prevalent post-THA, affecting about 2.2–2.4% patients who underwent the anterior approach [11,12,13]. Mounting evidence has supported the effectiveness of eccentric exercise along with heavy slow resistance when dealing with tendinopathy in assisting with the remodeling of the collagen fibers within the tendon [14]. Isometrics, particularly early on in the treatment of tendonitis, have also been shown to improve symptoms, but in reality, any type of mechanical loading of the tendon should create improvement [15]. This has been extensively researched in both Achilles and patellar tendinopathy, two of the most common tendinopathies, and can be extrapolated and applied to tendinopathies in other areas. Additionally, individuals participating in higher running speeds have been found to grapple with iliopsoas tendinopathy, with case studies showcasing successful rehabilitation through eccentric loading regimens [16]. The significance of iliopsoas strengthening in sprinters is underscored by studies indicating enhanced speed and endurance with strengthened iliopsoas muscles [17]. Apart from high-speed running, strengthening the hip flexor can benefit those in sports such as soccer in order to increase power and velocity when kicking a ball [18]_._

Finally, the hip flexor muscles are often found to be atrophied in populations with hip joint pathology such as osteoarthritis [19]. This, in turn, leads to deficits in gait and overall function [19]. In fact, this population was shown to be 50% slower in the stair climb test in a study out of the University of Colorado [20]. Thus, an exercise progression targeting the iliopsoas could benefit this demographic. One Australian study utilized magnetic resonance imaging (MRI) to measure the size of the iliopsoas on healthy people and found it to be a valid tool [19]. However, MRI is obviously quite an expensive tool to use. Therefore, the article suggests use of diagnostic ultrasound, which is becoming more readily available to physical therapists, to monitor the size of muscles such as the iliopsoas. This would allow for a more objective measure to rely on to visualize whether a change in muscle hypertrophy truly does occur throughout the application of the clinical exercise progression [19]. Clearly, a streamlined approach to iliopsoas strengthening would prove beneficial and applicable across multiple demographics.

While systematic reviews have extensively delved into methods for strengthening the gluteal muscles (Ebert et al., Moore et al.), the body of research concerning strengthening and activating the anterior hip musculature remains notably limited. In fact, several of the studies included in this review attempted to target core musculature and took EMG measurements of the anterior hip because they were looking for exercises that decrease hip flexor activation [21,22]. Seemingly few studies to date have focused on identifying effective methods on how to increase the activation of the hip flexors. This knowledge gap leaves clinicians, particularly those working with the aforementioned populations, relying on a trial-and-error approach in regard to exercise prescription and muscle specificity of the anterior hip. By gaining insights into which exercises effectively engage the iliopsoas muscle, clinicians can tailor interventions to directly address the muscle’s role as an anterior dynamic stabilizer, rather than employing a generic approach to strengthening the entire anterior hip musculature. For example, when looking to strengthen the gluteus medius muscle, clinicians can look to sources such as Moore, 2020, and Ebert, 2017, to guide them through what may be the best exercises to choose and how to progress them appropriately. Just as the physical therapy field has prioritized specificity in addressing issues related to the posterior aspect of the hip, the anterior aspect warrants equivalent attention to detail and targeted interventions.

As mentioned, the goal of this systematic review is to describe iliopsoas activation levels during rehabilitation exercises commonly used in a clinical setting and present an exercise progression for strengthening the iliopsoas based on EMG studies. While exercises such as the ASLR are commonly used to target the hip flexors, limited research specifies which muscles within the hip flexors are activated and at what phase of the movement. Moreover, no existing exercise progressions address low-level activation exercises through to high-level strengthening exercises for the hip flexors, particularly the iliopsoas. This information will enable clinicians to enhance their practice with specific populations experiencing hip micro-instability and dysplasia through exercise specificity.

## 2. Materials and Methods

The systematic review follows The Preferred Reporting Items for Systematic Review and Meta-Analysis (PRSIMA) guidelines as suggested in Liberati 2009 and Swartz 2011 An a priori protocol was completed according with PROSPERO guidelines and was registered on the PROSPERO website prior to submission for publication (registration number: CRD42024556236) [23,24].

### 2.1. Study Identification and Search Strategy

**Figure 1:**
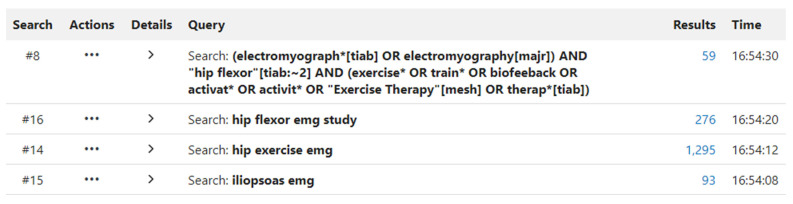
PubMed search strategy.

**Figure A1:**
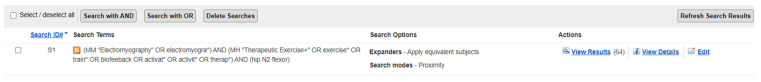
CINAHL Search Strategy.

**Figure A2:**
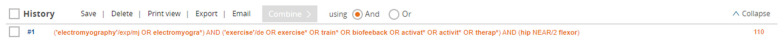
Embase search strategy.

Applicable articles were found by searching PubMed, CINAHL, and EMBASE databases in January 2024. The search strategy was overseen by a medical school librarian who facilitated the correct use of Boolean modifiers and appropriate translation of the search strategy across databases and ensured accuracy of the search based upon the study’s stated purpose. The keywords used were variations and derivatives of “electromyography”, “iliopsoas”, and “exercise therapy”. Figure 1 demonstrates the search strategy utilized for PubMed along with the correlated results. The search strategies used for CINAHL, and EMBASE are shown in Appendix A, Figure A1 and Figure A2. Certain articles that were identified through this process or by reviewing references of the articles that met the inclusion and exclusion criteria were included as well.

Additionally, to ensure a comprehensive identification process, hand-selected articles that were identified through the study selection process or by scouring the references of the included articles were also included.

### 2.2. Eligibility Criteria

The research question used to frame this systematic review outlined in Table 1 was as follows: which hip exercises have the greatest activation of the hip flexor muscles in a healthy population?

### 2.3. Study Selection

The search results of the various databases were put together, with duplicates deleted and filtered independently according to the specified inclusion and exclusion criteria by two members of the research team (Author 1: JJ., Author 2: KK.) using a citation manager, Zotero (Corporation of Digital Scholarship), and systematic review software management system, Covidence (Veritas Health Innovation, Melbourne, Australia). Discrepancies in the filtering of the search results were discussed by the two independent reviewers (Author 1: JJ, Author 2: KK). When the reviewers could not come to an agreement over these discrepancies, an a priori identified third member of the research team helped resolved the issue (Author 4: MJ).

### 2.4. Data Extraction

Data elements of the full-text articles were created based upon the question posed and the purpose of the current study. This included the types of exercises performed within the studies as well as the measurement of muscular activation such as percent of maximum volitional isometric contraction (MVIC), EMG amplitude, or RMS values.

### 2.5. Summary Measures and Synthesis of Results

The results were synthesized into three different tables, one for each form of EMG measurement: percent MVIC, EMG amplitude, and RMS value. The findings synthesized compare EMG activation of the iliacus, psoas, and iliopsoas with specific exercises. To ensure ease of implementation into clinical practice and ecological application of the results, an exercise progression including both closed-chain isometrics and open-chain exercises will be proposed. This progression was created utilizing a combination of the levels of activation demonstrated through the EMG studies analyzed and clinical expertise. Starting with less irritable movements that involve using the iliopsoas as a stabilizer to exercises where the iliopsoas becomes a primary mover.

### 2.6. Risk of Bias Assessment

Consistent with the Cochrane Handbook (Higgins 2019), the risk of bias and quality appraisal of the included studies were assessed [25]. The risk of bias assessment (RoB) of included studies was performed using the Modified Downs and Black Checklist for clinical trials. The Modified Downs and Black Checklist assessment was performed by the primary author and an independent research member (Author 1: JJ, Author 4: MJ, respectively), and the assessment outcomes were double-checked by a third member of the research team (Author 3: GL). Any discrepancies identified by the secondary review were clarified by an a priori identified third member of the research team.

## 3. Results

### 3.1. Study Selection and Characteristics

**Figure 2:**
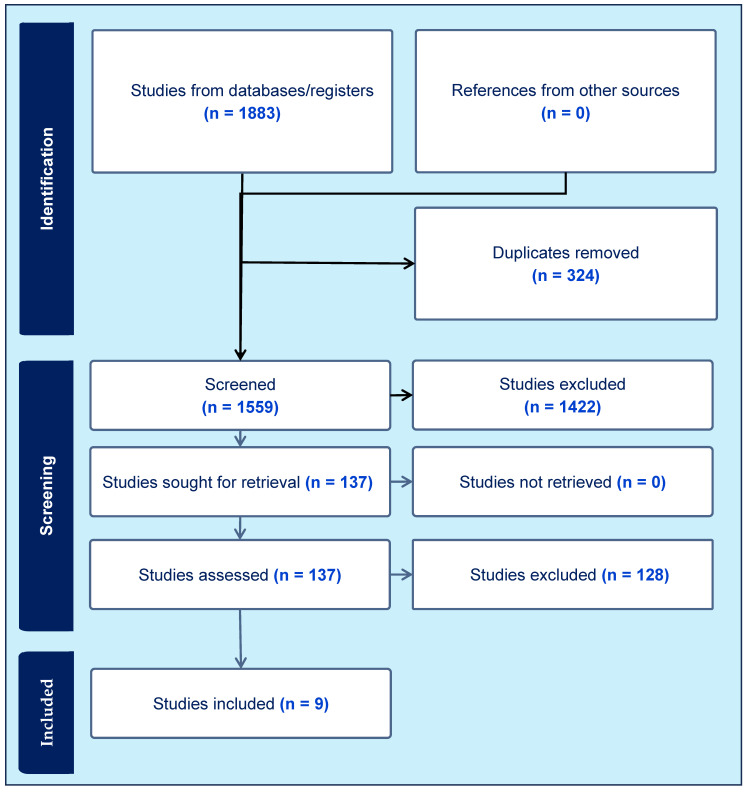
The PRISMA flow diagram.

The initial aggregate search results identified 1559 unique articles. Of the 137 articles read in full, 9 articles were deemed appropriate for final analysis. Six were cross-sectional studies, two were non-randomized crossover trials, and one was a descriptive laboratory study. A summary of the outcome characteristics is provided in Appendix B. Study characteristics included authors, study type, research question, patient population, methodology, and conclusions. Figure 2 outlines the study selection process in a PRISMA flow diagram and Table 2 describes the characteristics of each selected study in detail.

### 3.2. Risk of Bias Assessment

The Modified Downs and Black Checklist results for clinical trials are summarized in Table 3. The Modified Downs and Black Checklist assessment results for each individual study are provided in Appendix C. Andersson (1997), Jiroumaru (2014), Kim (2016), and Okubo (2021) received the highest risk of bias with a score of 13 and Philippon (2011) scored 14 on the checklist, which, according to the checklist, qualifies as “poor” (see Table 3) [26,28,29,31,33]. Andersson (1995), Hu (2011), Sugajima (1996) and Yamane (2019) scored 15, which qualifies as “fair” [27,30,32,34]. However, it is important to note that some of the categories where 0 points were given did not apply to the type of studies, such as blinding of the subjects. The lack of blinding in the rehabilitation and physical therapy literature is well documented and the Modified Downs and Black Checklist results in this review further corroborate this limitation (Armijo-Olivo, S. 2017) [35]. Studies that were found to have a “poor” risk of bias assessment were not excluded; however, Table 4 does outline which studies may be more reliable to pull data from and which were interpreted with more caution.

### 3.3. Summary Measures and Synthesis of Results

**Figure 3:**
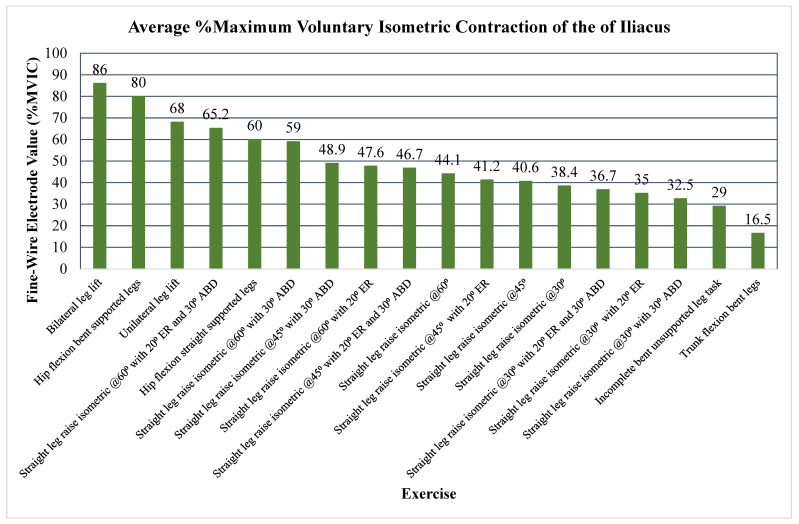
Average %MVIC activation of the iliacus. Note. ASLR; active straight leg raise; ABD, abduction; EMG, electromyography; ER, external rotation; MVIC, maximum volitional isometric contraction.

**Figure 4:**
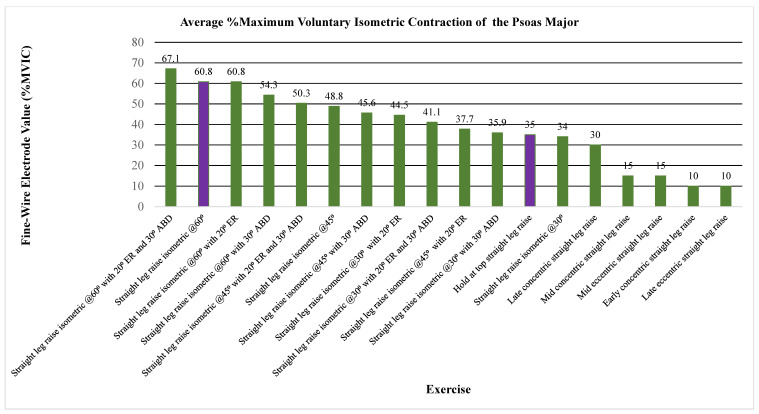
Average %MVIC Activation of the psoas major. Note. ASLR; active straight leg raise; ABD, abduction; EMG, electromyography; ER, external rotation; MVIC, maximum volitional isometric contraction. Purple bars highlight same exercises measured in different papers, a large difference can be seen despite the exercise being the same.

**Figure 5:**
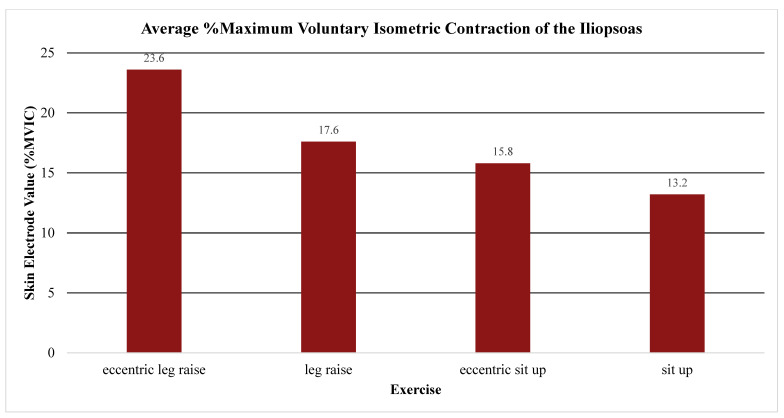
Average %MVIC activation of the iliopsoas. Note. ASLR; active straight leg raise; EMG, electromyography; MVIC, maximal volitional isometric contraction.

**Figure 6:**
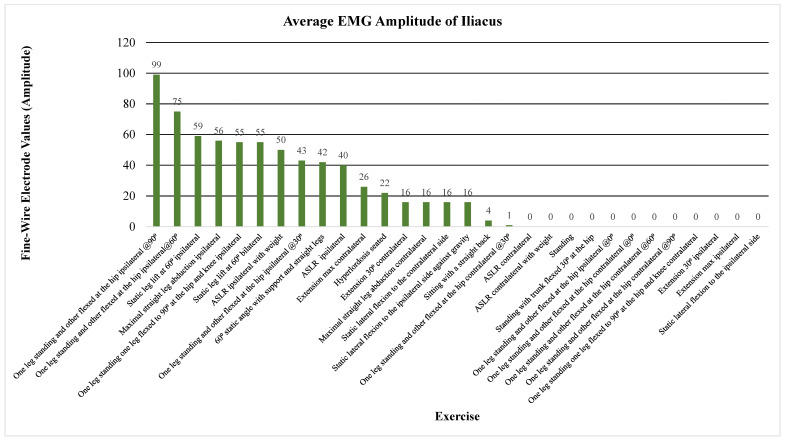
Average EMG amplitude of the iliacus. Note. ASLR; active straight leg raise; EMG, electromyography.

**Figure 7:**
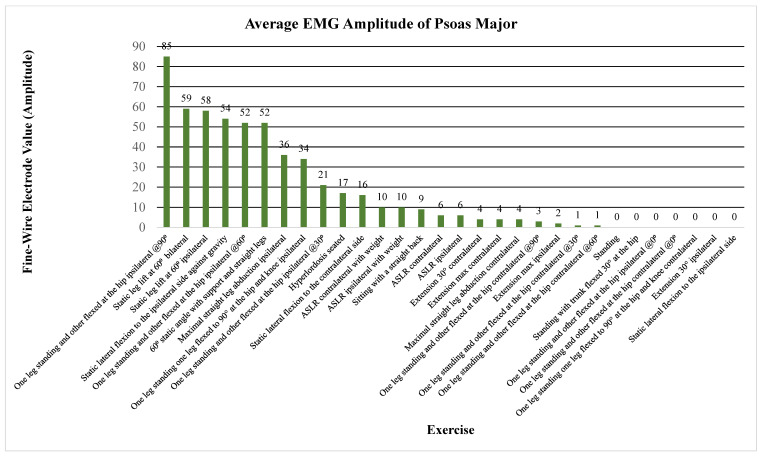
Average EMG amplitude of the psoas major. Note. ASLR; active straight leg raise; EMG, electromyography.

**Figure 8:**
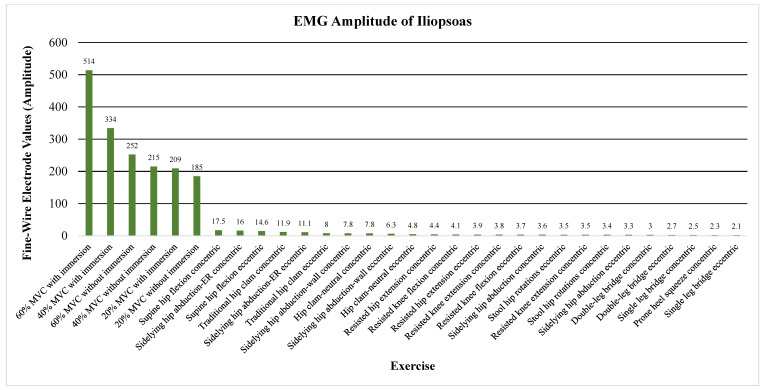
Average EMG amplitude of the iliopsoas. Note. EMG, electromyograph; ER, external rotation; MVC, maximum volitional contraction.

**Figure 9:**
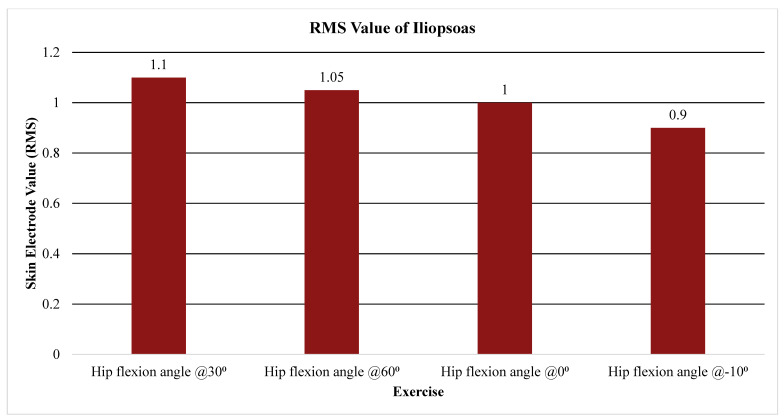
Average RMS value of the iliopsoas. Note. RMS, root mean square.

The primary outcome measure assessed the level of activation of the psoas, iliacus, or iliopsoas measured through percent MVIC (Andersson (1997), Okubo (2021), Yamane (2019), and Kim (2016)), amplitude (Sugajima (1996), Hu (2011), Andersson (1995), and Philippon (2011)), or root mean squared of the EMG from the max voluntary contraction (Jiroumaru (2014)) [26,27,28,29,30,31,32,33,34]. Measurements were conducted using either a fine-wire electrode (Andersson (1997), Okubo (2021), Yamane (2019), Sugajima (1996), Hu (2011), Andersson (1995), and (Philippon (2011)) or a surface electrode (Kim (2016) and Jiroumaru (2014)) while performing a specific exercise. Unless otherwise specified throughout the discussion, it can be assumed that fine-wire electrodes were used for the values mentioned (see Appendix D for specifics on which studies used which type of electrodes). Figure 3, Figure 4, Figure 5, Figure 6, Figure 7, Figure 8 and Figure 9 graphically display the results from each included study.

Results consistently showed increased activation of the iliacus, psoas, and iliopsoas during greater ranges of hip flexion, movement of the lower extremities on the trunk, trunk movement on the lower extremities while supported on the ground surface, and with added external resistance. The iliacus and psoas exhibited activation ranging from 44.1 to 65.2% MVIC and 35 to 67.1% MVIC, respectively, during greater degrees of hip flexion (See Figure 3, Figure 4 and Figure 5) [30].

Beginning with the most commonly included exercise, the straight leg raise, the iliacus demonstrated amplitudes of 40 µV and 50 µV without and with weight, respectively [27]. Meanwhile, the psoas showed amplitudes of 6 µV and 10 µV under the same conditions [27]. The highest activation of the ASLR was in 20° of external rotation and 30° of abduction in 60° of hip flexion according to Yamane et al. Amplitude values during a static leg lift at 60° resulted in 59 µV for a unilateral lift and 55 µV for a bilateral lift for the iliacus, compared to 58 µV for a unilateral lift with a comparable amount for the psoas [32]. At 90° of hip flexion in standing, the iliacus had an amplitude of 99 µV whereas the psoas had an amplitude of 85 µV [32]. See Figure 6 and Figure 7 for amplitude values of the iliacus and psoas during these exercises.

In regard to alternative exercises, refer to Figure 3, where the iliacus shows a high %MVIC during hip flexion with a straight trunk and the feet supported down at the ground (80% MVIC), bilateral lower extremity movement on the trunk (86% MVIC), and unilateral leg movement (68% MVIC) [26]. A movement that significantly activated the iliacus, not involving hip or trunk flexion, was maximal straight leg abduction with an amplitude of 56 µV (see Figure 6) [32]. As for the psoas, a notable exercise that activated the muscle significantly was static ipsilateral lateral trunk flexion against gravity, with an amplitude of 54 µV (see Figure 7) [32].

The combined iliopsoas showed substantial activation during supine hip flexion both concentrically (amplitude of 17.5 µV) and eccentrically (amplitude of 14.6 µV) as seen in Figure 8 [33]. It followed previously mentioned activation patterns in side-lying hip abduction (with some hip external rotation in this condition), with an amplitude of 16 µV (see Figure 8) [33]. It also portrayed activation patterns measured through skin electrodes similar to those of the iliacus and psoas individually, with RMS values of 1.1 and 1.05 at 30 and 60° of hip flexion, respectively (see Figure 9) [28].

Finally, all conditions tested with external load demonstrated increased activation, whether through added weight or water immersion. Particularly, the iliopsoas showed an increase in amplitude from 252 µV of amplitude–frequency to 514 µV when performing 60% MVC hip flexion contraction under water (see Figure 8) [34]. Appendix E demonstrates all individual exercises with their respective recorded EMG values.

## 4. Discussion

The purpose of this systematic review was to determine the amount of iliopsoas activation during common rehabilitation exercises. A secondary goal was to make the results immediately applicable to clinical setting by proposing a structured treatment progression based on the results. Across the nine included studies, methods to determine iliacus, psoas, or iliopsoas activation varied, including both fine-wire electrodes and surface EMG via adhesive electrodes. The muscle EMG was analyzed across a total of 135 exercises, with the most common exercises being the ASLR, sit-ups, and leg lowering. Variations of these and other exercise were also considered with different lower extremity and trunk positions, with and without external loads and with water resistance.

Several conclusions can be drawn from the results of this systematic review, which include the following:

(1) The iliopsoas can be activated in movements that involve stability of the spine and pelvis such as lateral trunk flexion against gravity or side-lying hip abduction. This suggests that the iliopsoas has an active role in lumbopelvic stability, evident through its activation in exercises not directly involving isotonic hip flexion or lateral trunk flexion. This included sitting with an upright trunk, the clamshell and side-lying hip abduction exercise, and resisted knee flexion and extension. Regarding the straight leg raise, the iliacus was largely active ipsilaterally, and quieter contralaterally, while the psoas was equally active both ipsilaterally and contralaterally. This potentially speaks to the psoas acting as more of a trunk stabilizer with this movement while the iliacus serves as the primary hip flexor or ipsilateral pelvic stabilizer.

(2) Moving the lower extremity on the spine (e.g., leg lowering versus moving the spine on the lower extremity with an exercise such as a sit-up) increased the activation of the iliopsoas. This is likely due to the active movement of the hip flexors required with active hip flexion, whereas subjects likely primarily used abdominal core musculature to perform a more classic version of a sit-up.

(3) Moving a longer lever during hip flexion in open-chain exercise (ASLR) will increase activation, particularly of the iliacus when compared to a short lever (supine hip flexion). This follows the principle of longer levers creating increased torque, therefore necessitating higher muscle activation to meet the demands of this increase [28].

(4) In closed-chain supine exercises such as straight spine hip flexion with the feet stabilized, a knee flexion posture resulted in greater activation of the iliopsoas than with the knees straight. This may be due to the hip flexors being at a more optimal biomechanical position to form a strong contraction than when extended such as in the supine position.

(5) Greater hip flexion angles in an ASLR (30–60 degrees) created higher activation levels of the iliopsoas than the 0–30 degree arc of motion. According to Jiroumaru et al., this is because the activation from other muscles such as tensor fascia latae and sartorius decreases in these ranges, and therefore the relative contribution of the iliopsoas increases [28].

(6) Bilateral movements such as bilateral leg lowering will cause increased activation, likely due to the need for increased stability.

(7) Adding resistance to exercises will increase muscle activation of those involved in producing a hip flexion movement.

The seven conclusive statements of the results listed above as well as the EMG results from the different exercises across the included studies were used to translate the results into a clinically friendly exercise progression targeting the iliopsoas. The intent of this review is to fill the gap created by the limited research specifically focused on strengthening of the hip flexors and to offer clinicians an evidence-based progression to follow when strengthening and training the anterior hip. A targeted approach to the iliopsoas can promote not only muscle strength (i.e., peak force output), but also the important ability to stabilize the femoral head while minimizing compensatory activation of muscles such as the tensor fascia latae. The role of stabilization is of particular importance with the aforementioned populations of hip dysplasia and micro-instability, where the iliopsoas plays a crucial role in the overall stability of the anterior joint. There are certainly other clinical patient demographics in which targeted, incremental loading of the iliopsoas would be indicated and who may also benefit from the proposed clinical progression. Such populations include patients seen post-total-hip-arthroplasty, athletic populations requiring rapid hip flexion (i.e., higher-speed running >7 m/s, persons diagnosed with persistent low back pain, coxa saltans (i.e., snapping hip syndrome), those with peripheral nerve injuries involving femoral nerve and/or nerve roots L1-3, and even post-partum individuals or those with pelvic floor dysfunction [36,37,38].

### 4.1. Risk of Bias

The nine studies included were assessed for risk of bias using the Modified Downs and Black Checklist (see Appendix C). The checklist provides 27 categories that can be responded to with a “yes”, no”, and with the responses “partially” or “unable to determine” for some items. Each “yes” response counts as a point, a point being positive in terms of decreasing the risk of bias, versus 0 points for a “no” response. All of the studies included in this systematic review scored between 13 and 15 points ranging from poor to fair risk of bias based on the checklist. The “no” responses were often under categories that were not relevant for the studies. For example, none of the studies included blinded subjects. With the type of EMG measurement used in the cross-sectional studies in this systematic review, it would have been unrealistic to blind the subjects. Overall, the scores of 13–15 are a small range, and the studies were deemed to have a similar risk of bias.

### 4.2. Comparison to Other Systematic Reviews

While countless systematic reviews analyze activation through EMG studies of the posterolateral hip, the author is not aware of any that they analyzed extensive data on the anterior hip. The methods in this systematic review mirror those that have been performed on the posterolateral hip (Moore 2020 and Ebert, 2017), including following the PRISMA guidelines, being conducted on homogenous patient populations, searching similar databases, using similar inclusion and exclusion criteria, performing a quality assessment, and using equivalent data extraction and analysis methods [21,22]. Regarding the anterior hip, one review looked at hip muscle activation in subjects with and without symptoms, but only one study looked at the iliacus or iliocapsularis [39]. A separate review looked at the effects of stretching the hip flexors on performance parameters but did not look at hip flexor strengthening [40]. Therefore, comparison to previous results from other similar systematic reviews was not possible, and more research needs to be performed regarding clinical implications of hip flexor strengthening and the utility of EMG within this research.

### 4.3. EMG Clinical Utility and Application

To ensure accurate interpretation and clinical application of the EMG results, the lead author consulted Dr. Joyce Campbell PT, PhD, EN, KEMG, an expert within the field of EMG and Director of The Electrophysiology Measurement Laboratory at California State University Long Beach. Through this exchange, Dr. Campbell described several key principles and limitations, pulling from her own knowledge and expertise along with information from the seminal Deluca article; these limitations were synthesized with the author of this systematic review below. They are imperative to consider when applying EMG results to clinical practice [41].

1. Volume Conduction and Motion Artifact: When using skin electrodes, all electrical signals below 400 Hz coming to the skin will be included. All frequencies above 400 Hz are not seen in the EMG signal; therefore, no fast glycolytic motor unit activity will be recorded. It is also impossible to identify the specific muscle(s) of origin (or separate out-motion electrical artifacts). As for the intramuscular electrodes, if the default low-cut filter is 20 Hz, even these will record contaminating cross-talk/volume conduction as muscle EMG. There should be some evidence of selecting a higher low-cut filter and/or repeating analysis with more selective filters to determine if the EMG conclusions would be improved.
2. Electrode Placement: The exact location of the placement of the electrodes influences the readings. If you are in the part of the muscle with a high concentration of motor units, the recording will reflect this. However, if the electrodes are placed, for example, near a fascial plane, the recording will not be as good. Depth also matters! Fast glycolytic muscle fibers tend to be more superficial, and slow oxidative fibers are deeper, so knowing the depth you are placing the electrode at is important. Furthermore, if the electrodes are taken out an any point, it will be impossible to re-create the same values, as the electrodes will never be in the exact same positioning.
3. Timing of Sample: The importance of beginning the recording of the sample prior to the subject even beginning the desired movement cannot be understated. This is because, often, the peak EMG happens so quickly (within milliseconds) that if the reading is taken too late, the peak value may actually be missed. It is also vital to begin the reading prior to movement in order to record the actual change in activation from the muscle at rest to the muscle in movement.
4. Heterogeneity in EMG Methodology: There are large inconsistencies from one study to the next whether we use the bandpass filter, intramuscular versus skin electrodes, reported outcome measurements in %MVIC, amplitude, or RMS of the EMG, electrode placement, and the timing of the sampling. This causes difficulty in comparing the studies and creates a need for extra scrutiny when evaluating the conclusion of each study.
5. Lack of Signal Normalization: Normalization of the EMG relying on an individual’s maximum effort on the day of testing is vital to compare values between subjects.
6. Erroneous EMG Extrapolations to Muscle Force: EMG does not predict muscle force production. Essentially, when there is change in velocity within a movement, there is no linear relationship between EMG and force output.

In discussion with Dr. Campbell, it is clear that within the physical therapy research, EMG studies are often misinterpreted, and the profession needs improvement as a whole in terms of the analysis and application of these studies. Refer to Table 4 created in collaboration for more specifics on how these limitations apply to the nine articles included in this review

### 4.4. Clinical Exercise Progression

The outlined progression in Appendix B with the associated table with figures was based on findings from this review, and we considered them alongside practice-based evidence and the author’s clinical expertise. It is important to note that the progression is meant as a guideline rather than a prescription and that it should be modified as needed for each individual. There is also a need for the progression to be validated in subsequent clinical trials to determine its true efficacy as well as its ecological and external validity.

The first phase begins with the implementation of very-low-level activation exercises that then transition into the second phase, including exercises that use the iliopsoas indirectly as a stabilizer but not as a primary mover. Following this phase, more direct activation of the iliopsoas is involved with the use of short progressing to long lever isometrics. Although the studies included have expressed that the iliopsoas is generally higher in activation in later ranges of hip flexion, the isometric progression in the fourth phase started at 60° and progressed towards 0°. This was the chosen order of the exercises because although there is more activation of the iliopsoas at 60°, and therefore starting there may be counterintuitive, there is less contribution from muscles such as the tensor fascia latae and the sartorius at this larger angle. The subject would then be able to gain the benefits of strengthening at a position where there is less activation of other accessory muscles, and then move into a position where there is a larger co-contraction once the iliopsoas has been strengthened on a more individual basis. The fifth phase begins the isotonic movements with the similar pattern of a short to long lever progression along with the first introduction of external load. Finally, in the sixth and seventh phase, eccentric movement as well as bilateral lower extremity movement is integrated into the progression for the highest level of iliopsoas strength training. While the recommended dosage is included in this progression, it is up to the clinician’s discretion to adjust the exercises and dosage to each individual patient as appropriate. A criterion for progression from each phase is included for the clinician’s reference as well.

### 4.5. Limitations

A notable limitation of this systematic review is that all studies were performed on healthy subjects, necessitating caution when applying findings to populations with hip pathology. Furthermore, variation in EMG measurement methods and exercise protocols across studies posed challenges in direct comparisons and exercise progression formulation. For example, Okubo (2021) performed an isometric hold at “the top of the straight leg raise” and achieved 35% MVIC activation of the iliopsoas, while Yamane (2019) reached 60.8% MVIC with an isometric hold at 60° of an ASLR [29,30]. The difference can likely be attributed to the methodology of EMG instrumentation and measurement or the setup and execution of the exercise. Due to the fact that any one of the multiple discrepancies that exist across the methods of these two studies (and the other included study) could explain the difference in the resultant EMG, identification of which specific independent variable was responsible for different results was difficult due to confounding variables. Lastly, the process of putting together an exercise progression, based upon the studies included, required practiced-based evidence and clinical expertise from individuals other than the authors. For example, if going solely based on activation levels, some isometric exercises would be put after something such as a weighted open chain exercise. However, concepts such as consideration of the length of the lever being moved as well as the amount of additional torque required by adding external loading were considered. It is also important to note that the EMG studies utilized to create this progression influenced the choice of exercises by giving guidance to which exercises the iliopsoas is most active with, and this does not directly correlate to indications of the force output of the muscle. This review also does not take into account the timing of muscle activation onset, which can be influential on the function of the muscle itself.

## 5. Conclusions

In conclusion, while research regarding training the iliopsoas is limited, this review provides practitioners with a specific progression to follow based on the existing evidence. The current systematic review cohesively describes the most current literature in regard to iliopsoas activation patterns with specific exercise. Future research should focus on analyzing a larger breadth of rehabilitation exercises regarding iliopsoas strengthening and activation. There should also be further research conducted utilizing populations with diagnoses of hip dysplasia or hip micro-instability to determine whether activation patterns may be different for this population as well as how the stability of the femoral head changes with increased iliopsoas strength and activation.

Several limitations and the misinterpretations of EMG results exist in both the literature and clinical practice to date. To avoid erroneous conclusions and to improve the accuracy of the translational EMG science, it is recommended that future researchers consider ensuring that the best practices are used in their study designs and ensuring the consistency of anatomical electrode placement, proper signal filtering, unanimous use of intramuscular EMG, and control of the practitioner dependent variables that influence EMG results. Moreover, future systematic reviews that seek to provide clinical recommendations of exercise selection and prescription based upon EMG data should be intentional in their inclusion and exclusion criteria to filter primary studies that use surface EMG and/or have fatal limitations in their methodology that would preclude the results from having external validity and applicability to clinical practice.

In hopes of facilitating the immediate application of the current findings into clinical practice and for ease of translatability, the evidence-based progression is proposed. The progression follows the principle of incremental and progressive overload, initiating with low-level activation exercise through static posture and isometrics and progressing to closed-chain exercise, open-chained short lever exercise, and finally long lever open-chain exercise without and with external resistance.

## Figures and Tables

**Table 1:** Question and study design inclusion and exclusion criteria.

Question Component	Inclusion Criteria	Exclusion Criteria
Population	Healthy (no comorbidities, no history of low back/hip pain)	Non-human study, cadaver study, pathological population
Intervention	E EMG study of the hip flexors	No EMG measurement of iliopsoas specifically
Comparison	• n/a	• n/a
Outcome	• n/a	• n/a
Study Design	• any	• any
Time	• any	• any

Note. N/A: indicates information not applicable; EMG: electromyography.

**Table 2:** Characteristics of Included Studies.

First Author (Year)	Study Type	Research Question(s)/Hypotheses	Patient Population Specifics	Methodology	Conclusions
Andersson, 1997 [26]	Cross- sectional study	Compare EMG levels in sub-maximal training exercises for the trunk and hip flexor muscles with those voluntarily attainable in corresponding situations.	N = 6 healthy Average age: 25 (22–29) year Body mass: 75 (65–84) kg Height: 1.81 (1.76–1.87) m	Measurement tool: Indwelling for IL: 3 cm lateral to the femoral artery, 1 cm medial to the SA muscle and 1 cm inferior to the inguinal ligament. Mode: On a horizontal bench performing trunk or leg lift-based exercises Primary outcome measure: %MVIC	Exercise in knee flexed or unsupported LE for hip flexor activation. Bent unsupported legs will activate iliopsoas in early trunk flexion. EMG values are higher in concentric mode.
Hu, 2011 [27]	Non-randomized crossover trial	During hip flexion, does the psoas always have the same function as the iliacus? Does the psoas affect the hip more than the lumbar spine (is the psoas a hip flexor in a SLR)?	N = 17 healthy females Age: 28.7 ± 2.8 years Weight: 60.7 ± 9.7 kg Height: 167.6 ± 7.5 cm BMI: 21.5 ± 2.4	Measurement tool: Fine wire for IL iliacus insertion was 1 cm inferior to the inguinal ligament, 2 cm medial to a vertical line down from the anterior superior iliac spine, and for the psoas major, 5–8 cm lateral to L3–L4 Mode: Supine, straight leg, foot DF. Raise leg 3 × 20 cm above table keep leg elevated 10s, again with weight. Primary outcome measure: Amplitude	Psoas acting primarily as a trunk stabilizer in SLR. Iliacus does work as a hip flexor. More activation with weight.
Jiroumaru, 2014 [28]	Cross-sectional study	What are individual muscle contributions to hip flexion torque?	N = 10 healthy males Age: 27.2 ± 2.7 years Weight: 67.2 ± 6.3 kg Height: 172.0 ± 3.8 cm Subgroup of six subjects underwent further MRI measurements Age: 28.7 ± 1.8 years Weight: 69.3 ± 7.1 kg Height: 171.2 ± 3.9 cm	Measurement tool: Skin electrode for IP 3–5 cm distal from the ASIS Mode: Isometric hip flexion at 0°, 30°, 60°, 90°. Primary outcome measure: Root mean square value	Muscle length of the IL scarcely changed, little change in EMG activity. This suggests that contribution from IL increases as angle of hip flexion increases.
Okubo, 2021 [29]	Cross-sectional study	What is the activation of the hip flexor and abdominal muscles during an ASLR to end-range hip flexion?	N = 18 healthy males Age: 25 ± 4 years Height: 170.1 ± 6.2 cm Body mass: 60.3 ± 4.7 kg Exclusion criteria: previous low back pain, spinal surgery, lower extremity surgery or any neurological disorder. Data for nine participants were excluded	Measurement tool: Fine wire: PM electrode was inserted 1.5 cm lateral to the transversus process to a depth of 3 cm ventral to the border between erector spinae and quadratus lumborum at an angle of 20° to the sagittal plane Mode: Concentric, hold (at end range) and eccentric phases of an ASLR. Primary outcome measure: %MVIC	PM activated prior to leg movement. During the ASLR, PM EMG continues to increase towards the end range of hip flexion. PM EMG was greater in the late elevation phase of the ASLR and the holding phase.
Yamane, 2019 [30]	Cross-sectional study	Elucidate the activities of the hip flexor muscles during straight leg raising (SLR) in healthy subjects. Also investigated the activities of these muscles during SLR with deep flexion, abduction, and external rotation.	N = 10 healthy males Age: 21.1 ± 1.0 years Height:174.7 ± 5.0 cm, weight:, 66.8 ± 6.5 kg Body mass index: 21.9 ± 1.5 kg/m^2^. Excluded if they had a history of lumbar, pelvis, hip, or lower extremity disease; neurological deficit; a physical condition with a passive SLR angle of <60°.	Measurement tool: Fine wire: PM inserted to a depth of 7–9 cm from the skin via a route 7 cm lateral to the spinous process between the L3–L4 transverse process at an angle of 20° to the sagittal plane and IL 2 cm medial to the anterior superior iliac spine (ASIS), and 1 cm distal to the inguinal ligament Mode: SLR x12 @ 30°, 45°, and 60°; abduction @ 0° and 20°; and external rotation @ 0° and 30° Primary outcomes measure: %MVIC	SLR of up to 60° mainly activates PM and IL at larger hip flexion angles For constant hip flexion, the %MVC values for the PM and IL showed no significant changes with hip abduction and external rotation.
Kim, 2016 [31]	Cross-sectional study	Comparison of muscular activities in the abdomen and lower limbs while performing sit-ups and leg-raises.	N = 20 healthy Age: 20.5 years 8 males Age: 20.5 years Height: 173.4 cm Weight: 65.3 kg 12 females Height: 161.4 cm Weight: 53.4 kg	Measurement tool: Surface sensors; IP sensor was attached to the medial aspect of the rectus femoris, inferior to the inguinal ligament. Mode: Active straight leg raise Primary outcome measure: %MVIC	Straight leg raise is more appropriate for reinforcement of hip flexors since the activation of the flexors of the lower limbs is high
Andersson, 1995 [32]	Cross-Sectional study	What are the roles of the psoas and iliacus muscles in the straight leg raise?	N = 7 healthy 4 men with an average age: 32 (23) years, body mass: 85 (22) kg, and height: 1.89 (20.05) m 3 women, 28 (24) years, 64 (23) kg and 1.73 (0.05) m,	Measurement tool: Ultrasound-guided fine-wire electrodes IL 3 cm lateral to the femoral artery, 1 cm medial to the sartorius muscle and 1 cm inferior to the inguinal ligament, 3 cm deep from the skin surface and PM 5–8 cm lateral to the spinal processes, to a depth from the skin of between 8.5 and 12.5 cm. Mode: Various tasks were performed standing, sitting, and lying. Primary outcome measure: amplitude	In contralateral leg extension in standing, the function of IL is to stabilize between the pelvis and hip. PM would assist in stabilizing the lumbar spine when a heavy load is applied on the contralateral side. IL not activated. Under most conditions, PM and IL muscles showed a common activation pattern.
Philippon, 2011 [33]	Descriptive laboratory study	A progression of hip rehabilitation exercises to strengthen the gluteus medius muscle could be identified that minimize concurrent iliopsoas muscle activation to reduce the risk of developing or aggravating hip flexor tendinitis.	N = 10 healthy	Measurement tool: Ultrasound-guided fine-wire IP 2 finger-breadths lateral to the femoral artery and 1 fingerbreadth below the inguinal ligament. Mode: 13 hip rehabilitation exercises. Primary outcome measure: Amplitude	Outlined exercises to avoid when IP pain or tendinitis is a concern.
Sugajima, 1996 [34]	Non-randomized crossover trial	Long-term spaceflight resulted in disuse muscle atrophy. The principal cause of these changes is the removal of static load from the weight-bearing musculature. The unloading conditions mimicked the sudden effect that weightlessness in parabolic or space flight had on neuromuscular functions.	N = 11 healthy males Age, 23.3 + 2.0 years; height, 173.1 + 6.2 cm; body mass, 69.2 2 4.9 kg; body fat, 10.5 ± 1.1%; all values, means + SD	Measurement tool: Fine-wire electrodes IP at fleshy fibers near the hip joint Mode: Voluntary isometric contraction while the subjects were standing with hip on the test side flexed to 60° and to 120°. Primary outcome measure: Amplitude	Concluded that water immersion facilitated recruitment of larger motor units in IP.

Note. ASLR, active straight leg raise; BMI, body mass index; Cm, centimeters; DF, dorsiflexion; EMG, electromyography; Kg, kilograms; IL, iliacus; IP, iliopsoas; M, meters; MVIC, maximum voluntary isometric contraction; PM, psoas major.; s, seconds; SD, standard deviation; SLR, straight leg raise; Y, year.

**Table 3:** Summary of risk of bias assessment.

Downs and Black Criteria	Andersson 1997 [26]	Andersson 1995 [32]	Hu 2011 [27]	Jiroumaru 2014 [28]	Kim 2016 [31]	Okubo 2021 [29]	Philippon 2011 [33]	Sugajima 1996 [34]	Yamane 2019 [30]
1									
2									
3									
4									
5									
6									
7									
8									
9									
10									
11									
12									
13									
14									
15									
16									
17									
18									
19									
20									
21									
22									
23									
24									
25									
26									
27									
* Total	13	15	15	13	13	13	14	15	15

Note. Red cell indicates that criteria were not met or that we were unable to determine whether or not they were (0 points); green cell indicates that criteria were met (1 point); * total row indicates the aggregate number of points per column (i.e., for each article).

**Table 4:** EMG limitations across the nine studies.

First Author (Year)	Methodology	Strengths and Limitations	Interpretation of Analysis
Andersson (1995) [32]	Bandpass: 10–1000 Hz Sampling rate 0.5 kHz Sampling period: first 2 s after stable position achieved	Limitations: Low-cut/high-pass filter being at 10 Hz Sampling is conducted after the contraction is stabilized Strengths: The references and clinical discussion are accurate	Clinically applicable
Andersson (1997) [26]	Bandpass: 10–1000 Hz Limited signal processing information Sampling period: 200 ms before start of motion Intramuscular and skin EMG data analyzed as one group without acknowledgment of volume conduction	Limitations: Fallibility and limitations of skin electrodes not controlled for Skin electrode data being combined with the intramuscular data	Caution with clinical application
Hu (2011) [27]	Bandpass 20 Hz–1kHz Confusing information regarding their filtering process Sampling rate 2 kHz Analyzed median EMG for 3 s, 5–10 s after beginning of movement	Limitations: Problems in methodology Strengths: The documentation of psoas recruitment bilaterally does support its role in stabilization of the spine in the SLR test position	Clinically applicable
Jiroumaru (2014) [28]	Skin electrodes used No filters given Sampling rate: 1000 Hz EMG sampled over 2 s in the middle of sustained contraction	Limitations: Conclusions within paper cannot be substantiated by the methods used	Limited clinical applicability
Kim (2016) [31]	Skin electrodes used without recognition of volume conduction contamination. No filters given Sampling rate: 1500 Hz Analyzed middle 3 s of MVIC isometric	Limitations: Poor methodology and lack of consideration of skin movement No discussion on standard deviation of EMG from individual electrodes across the subjects	Caution with clinical application
Okubo (2021) [29]	20–500 Hz bandpass Sampling rate 1000 Hz Used algorithm to determine onset of EMG in ASLR	Limitations: Authors failed to consider limitations of the skin electrodes in the muscles other than the iliopsoas and how that affects the conclusions drawn The author was unaware that skin electrodes do not detect EMG during low intensity effort, and therefore cannot conclude anything on the onset of muscle recruitment Strengths: A 500 Hz high-cut filter for skin electrodes was used, which is acceptable as it will not include the skin frequencies, but is inadequate for the intramuscular electrodes	Surface EMG data: limited clinical applicability Intramuscular EMG data: clinically applicable
Philippon (2011) [33]	No bandpass provided Sampling rate: 1200 Hz No sample timing provided	Strengths: Relative recruitment of the iliopsoas within the one test session is useful to rank the exercises	Clinically applicable
Sugajima (1996) [34]	Bandpass 100 Hz–10 kHz Computerized motor unit analysis relying on “spike” Only a few motor units could be identified in the fine wire EMG Arbitrary designation of <500 µV as a low-amplitude unit versus >500 µV as a high-amplitude one	Limitations: The “spike” analysis—as EMG increased with increasing effort, there was superimposition of the motor unit action; this makes it impossible to assess individual spikes and causes error introduced by overlapping and summation of the motor unit action potentials Strengths: A shift in motor unit recruitment with immersion in water is intriguing	Caution with clinical application
Yamane (2019) [30]	Bandpass 20–500 Hz Sampling rate 1000 Hz	Limitations: Assumption is made that greater hip flexion torque is produced at 60° of hip flexion without measuring force No recognition of volume conduction or the fact that they filtered out all intramuscular EMG data above 500 Hz	Limited clinical applicability due to unvalidated assumptions that were used to create the conclusion

Note. EMG, electromyography; Hz, hertz; kHz, kilohertz, µV, microVolts; SLR, straight leg raise.

## Data Availability

MDPI is a member of COPE. We fully adhere to its Core Practices and to its Guidelines. MDPI journals uphold a rigorous peer review process together with clear ethical policies and standards to support the addition of high-quality scientific studies to the field of scholarly publication. Where we become aware of ethical issues, we are committed to investigating them and taking necessary action to maintain the integrity of the literature and ensure the safety of research participants.

## Appendix B. Iliopsoas Strengthening Progression

**Table A1:** Iliopsoas Strengthening Progression.

Phase	Level	Weight Bearing Status	Movement	Exercise	Parameters
**Activation—Stability Focused**	1	Non-Weight-Bearing	Static	Sitting with a straight back	5 × 45 s 3×/Day 7 Days/Week 1 Week
				Sitting in hyperlordosis	
	2	Non-Weight-Bearing	Dynamic	Side-lying traditional clam shell	3 × 10 each side 1×/Day 1 Week
			Dynamic	Side-lying hip abduction	
			Static	Seated lateral flexion of the trunk against gravity	3 × 45 s 1×/Day 1 Week
	3	Non-Weight-Bearing	Static	A. Seated isometric at 90° of hip flexion	5 × 45 s 1×/Day Progress from 25 to 100% MVIC 1 Week Exercise A 1 Week Exercise B
		Single-Limb Support		B. Standing ipsilateral hip flexion isometric at 30°	
	3+	Single-Limb Support	Static	A. Standing ipsilateral hip flexion isometric at 60°	5 × 45 s 3–4 Days/Week Progress from 25 to 100% MVIC 1 Week Exercise A 1 Week Exercise B
				B. Standing ipsilateral hip flexion isometric at 90°	
**Strength**	4	Non-Weight-Bearing	Static	A. Straight leg isometric at 60° of hip flexion	5 × 45 s 3–4 Days/Week Progress from 25 to 100% MVIC 1 Week Exercise A 1 Week Exercise B 1 Week Exercise C
				B. Straight leg isometric at 30° of hip flexion	
				C. Straight leg isometric at 0° of hip flexion	
	**5**	Non-Weight-Bearing	Dynamic	A. Supine hip flexion	3 × 12 3–4 Days/Week 1 Week Exercise A 1 Week Exercise B 1 Week Exercise C
				B. Active straight leg raise	
				C. Active straight leg raise with weight	
	**6**	Non-Weight-Bearing	Dynamic	A. Trunk flexion with bent knees	3 × 8 3–4 Days/Week 1 Week Exercise A 1 Week Exercise B 1 Week Exercise C
				B. Hip flexion with straight supported legs	
				C. Hip flexion with bent supported legs	
	**7**	Non-Weight-Bearing	Dynamic	A. Eccentric leg raise	3 × 8 3–4 Days/Week 1 Week Exercise A 1 Week Exercise B 1 Week Exercise C 1 week Exercise D
				B. Eccentric leg raise with weight	
				C. Bilateral leg lift	
				D. Bilateral leg lift with weight	

**Table A2:** Iliopsoas strengthening progression goals and criteria.

Level	Goal	Criteria for Progression
1	Low-level activation	❐ Able to hold complete sets and repetitions with good form ❐ No pain with exercise
2	Activation as an indirect stabilizing muscle	❐ Able to hold complete sets and repetitions with good form ❐ No pain with exercise
3/3+	Direct higher-level activation of the muscle	❐ Able to hold complete sets and repetitions with good form ❐ No pain with exercise ❐ Consistency with exercise
4	Beginning of strength phase—open-chain long lever isometric	❐ Able to hold complete sets and repetitions with good form ❐ No pain with exercise ❐ Consistency with exercise
5	Transition into isotonic strengthening	❐ Able to hold complete sets and repetitions with good form ❐ No pain with exercise ❐ Consistency with exercise
6	Strengthen in closed-chain isotonic table exercises	❐ Able to hold complete sets and repetitions with good form ❐ Able to differentiate between spine and hip flexion movement ❐ No pain with exercise ❐ Consistency with exercise
7	Incorporate eccentric movement and bilateral movements for higher intensity strengthening	❐ Able to hold complete sets and repetitions with good form ❐ No form break down with added external load ❐ No pain with exercise ❐ Consistency with exercise

**Table A3:** Iliopsoas Progression Exercise Images.

Exercise	Starting Position	Ending Position
Sitting with a straight back		
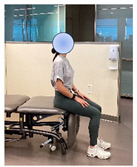
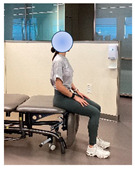
Sitting in hyperlordosis		
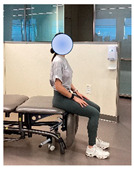
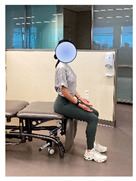
Side-lying traditional clam shell		
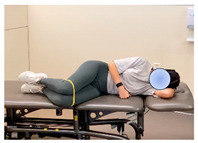
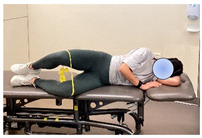
Side-lying hip abduction		
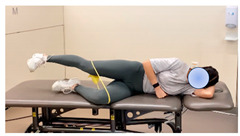
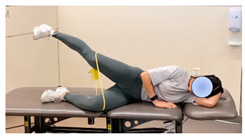
Seated lateral flexion of the trunk against gravity		
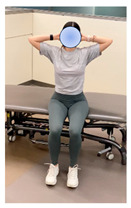
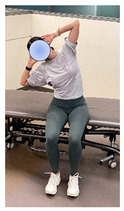
Seated isometric at 90° of hip flexion		
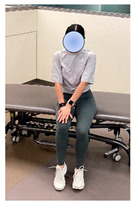
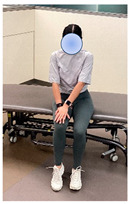
Standing ipsilateral hip flexion isometric at 30°		
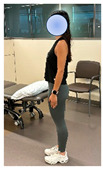
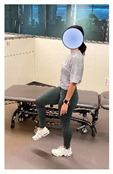
Standing ipsilateral hip flexion isometric at 60°		
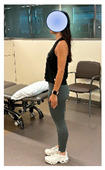
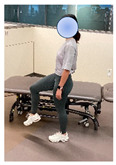
Standing ipsilateral hip flexion isometric at 90°		
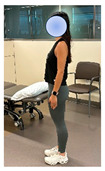
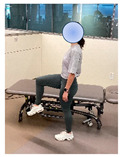
Straight leg isometric at 60° of hip flexion		
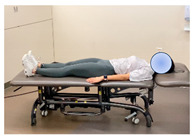
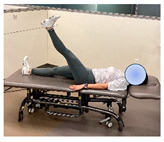
Straight leg isometric at 30° of hip flexion		
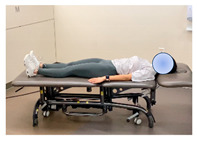
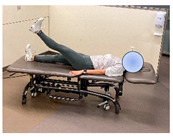
Straight leg isometric at 0° of hip flexion		
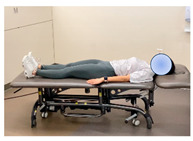
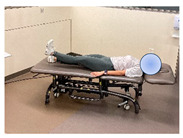
Supine hip flexion		
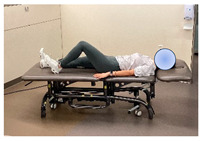
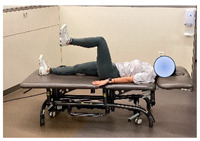
Active straight leg raise		
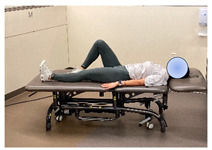
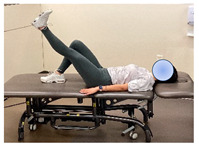
Active straight leg raise with weight		
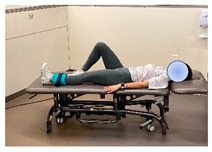
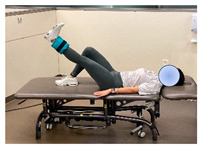
Trunk flexion with bent knees		
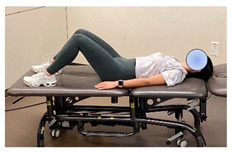
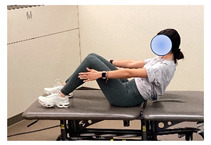
Hip flexion with straight supported legs		
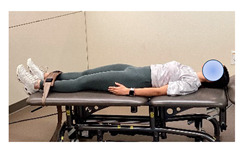
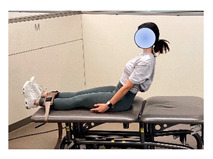
Hip flexion with bent supported legs		
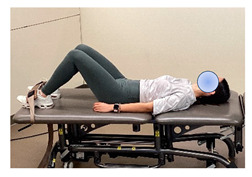
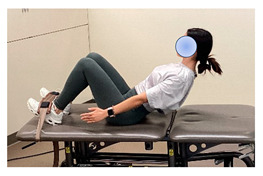
Eccentric leg raise		
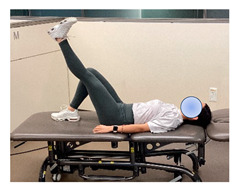
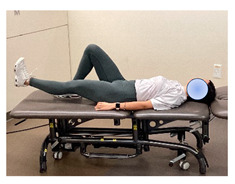
Eccentric leg raise with weight		
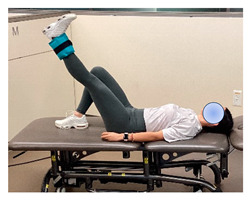
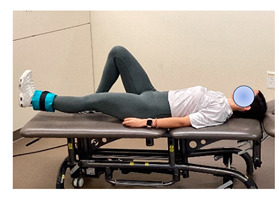
Bilateral leg lift		
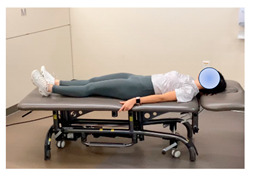
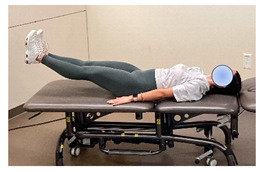
Bilateral leg lift with weight		
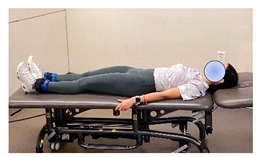
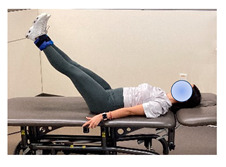

## Appendix C. Risk of Bias Tool

**Table A4:** Modified Downs and Black Checklist for risk of bias (RoB) assessment of non-randomized clinical trials.

Item	Criteria	Answers
**Reporting**		
1	*Is the hypothesis/aim/objective of the study clearly described?*	Yes = 1 No = 0
2	*Are the main outcomes to be measured clearly described in the Introduction or Methods section?* If the main outcomes are first mentioned in the Results section, the question should be answered no.	Yes = 1 No = 0
3	*Are the characteristics of the patients included in the study clearly described?* In cohort studies and trials, inclusion and/or exclusion criteria should be given. In case–control studies, a case definition and the source for controls should be given.	Yes = 1 No = 0
4	*Are the interventions of interest clearly described?* Treatments and placebo (where relevant) that are to be compared should be clearly described.	Yes = 1 No = 0
5	*Are the distributions of principal confounders in each group of subjects to be compared clearly described?* A list of principal confounders is provided.	Yes = 2 Partially = 1 No = 0
6	*Are the main findings of the study clearly described?* Simple outcome data (including denominators and numerators) should be reported for all major findings so that the reader can check the major analyses and conclusions. (This question does not cover statistical tests that are considered below).	Yes = 1 No = 0
7	*Does the study provide estimates of the random variability in the data for the main outcomes? * In non-normally distributed data, the interquartile range of results should be reported. In normally distributed data, the standard error, standard deviation or confidence intervals should be reported. If the distribution of the data are not described, it must be assumed that the estimates used were appropriate and the question should be answered “yes”.	Yes = 1 No = 0
8	*Have all important adverse events that may be a consequence of the intervention been reported? * This should be answered “yes” if the study demonstrates that there was a comprehensive attempt to measure adverse events. (A list of possible adverse events is provided).	Yes = 1 No = 0
9	*Have the characteristics of patients lost to follow-up been described? * This should be answered “yes” where there were no losses to follow-up or where losses to follow-up were so small that findings would be unaffected by their inclusion. This should be answered “no” where a study does not report the number of patients lost to follow-up.	Yes = 1 No = 0
10	*Have actual probability values been reported (*e.g., *0.035 rather than <0.05) for the main outcomes except where the probability value is less than 0.001?*	Yes = 1 No = 0
**External validity**		
11	*Were the subjects asked to participate in the study representative of the entire population from which they were recruited? * The study must identify the source population for patients and describe how the patients were selected. Patients would be representative if they comprised the entire source population, an unselected sample of consecutive patients, or a random sample. Random sampling is only feasible where a list of all members of the relevant population exists. Where a study does not report the proportion of the source population from which the patients are derived, the question should be answered as “unable to determine”.	Yes = 1 No = 0 Unable to determine = 0
12	*Were those subjects who were prepared to participate representative of the entire population from which they were recruited? * The proportion of those asked who agreed should be stated. Validation that the sample was representative would include demonstrating that the distribution of the main confounding factors was the same in the study sample and the source population.	Yes = 1 No = 0 Unable to determine = 0
13	*Were the staff, places, and facilities where the patients were treated, representative of the treatment the majority of patients receive? * For the question to be answered “yes”, the study should demonstrate that the intervention was representative of that in use in the source population. The question should be answered “no” if, for example, the intervention was undertaken in a specialist center unrepresentative of the hospitals most of the source population would attend.	Yes = 1 No = 0 Unable to determine = 0
**Internal validity—bias**		
14	*Was an attempt made to blind study subjects to the intervention they have received? * For studies where the patients would have no way of knowing which intervention they received, this should be answered “yes”.	Yes = 1 No = 0 Unable to determine = 0
15	*Was an attempt made to blind those measuring the main outcomes of the intervention?*	Yes = 1 No = 0 Unable to determine = 0
17	*In trials and cohort studies, do the analyses adjust for different lengths of follow-up of patients, or in case–control studies, is the time period between the intervention and outcome the same for cases and controls? * Where follow-up was the same for all study patients, the answer should be “yes”. If different lengths of follow-up were adjusted for by, for example, survival analysis, the answer should be “yes”. Studies where differences in follow-up are ignored should be answered “no”.	Yes = 1 No = 0 Unable to determine = 0
18	*Were the statistical tests used to assess the main outcomes appropriate? * The statistical techniques used must be appropriate to the data. For example, nonparametric methods should be used for small sample sizes. Where little statistical analysis has been undertaken but where there is no evidence of bias, the question should be answered “yes”. If the distribution of the data (normal or not) is not described, it must be assumed that the estimates used were appropriate and the question should be answered “yes”.	Yes = 1 No = 0 Unable to determine = 0
19	*Was compliance with the intervention/s reliable? * Where there was noncompliance with the allocated treatment or where there was contamination of one group, the question should be answered “no”. For studies where the effect of any misclassification was likely to bias any association to the null, the question should be answered “yes”.	Yes = 1 No = 0 Unable to determine = 0
20	*Were the main outcome measures used accurate (valid and reliable)? * For studies where the outcome measures are clearly described, the question should be answered “yes”. For studies which refer to other work or that demonstrates the outcome measures are accurate, the question should be answered as “yes”.	Yes = 1 No = 0 Unable to determine = 0
**Internal validity—confounding (selection bias)**		
21	*Were the patients in different intervention groups (trials and cohort studies) or were the cases and controls (case–control studies) recruited from the same population?* For example, patients for all comparison groups should be selected from the same hospital. The question should be answered “unable to determine” for cohort and case–control studies where there is no information concerning the source of patients included in the study.	Yes = 1 No = 0 Unable to determine = 0
22	*Were study subjects in different intervention groups (trials and cohort studies) or were the cases and controls (case–control studies) recruited over the same period of time?* For a study that does not specify the time period over which patients were recruited, the question should be answered as “unable to determine”.	Yes = 1 No = 0 Unable to determine = 0
23	*Were study subjects randomized to intervention groups?* Studies that state that subjects were randomized should be answered “yes” except where the method of randomization would not ensure random allocation. For example, alternate allocation would score “no” because it is predictable.	Yes = 1 No = 0 Unable to determine = 0
24	*Was the randomized intervention assignment concealed from both patients and health care staff until recruitment was complete and irrevocable?* All nonrandomized studies should be answered “no”. If assignment was concealed from patients but not from staff, it should be answered “no”.	Yes = 1 No = 0 Unable to determine = 0
25	*Was there adequate adjustment for confounding in the analyses from which the main findings were drawn? * This question should be answered “no” for trials if the main conclusions of the study were based on analyses of treatment rather than intention to treat; the distribution of known confounders in the different treatment groups was not described; or the distribution of known confounders differed between the treatment groups but was not taken into account in the analyses. In non-randomized studies, if the effect of the main confounders was not investigated or confounding was demonstrated but no adjustment was made in the final analyses, the question should be answered as “no”.	Yes = 1 No = 0 Unable to determine = 0
26	*Were losses of patients to follow-up taken into account? * If the numbers of patients lost to follow-up are not reported, the question should be answered as “unable to determine”. If the proportion lost to follow-up was too small to affect the main findings, the question should be answered “yes”.	Yes = 1 No = 0 Unable to determine = 0
**Power**		
27	*Was a power analysis performed?*	Yes = 1 No = 0 Unable to determine = 0

## Appendix D. Electrode Type

**Table A5:** Type of electrode used per study.

First Author/Year	Fine-Wire Electrode	Surface Electrode
Andersson et al., 1997 [26]	Fine-Wire Iiliacus Bandpass Filter 10–1000 Hz	
Okubo et al., 2021 [29]	Fine-Wire Psoas Major	
Yamane et al., 2019 [30]	Fine-wire Psoas Major and Iliacus	
Kim et al., 2016 [31]		Surface sensor iliopsoas
Sugajima et al., 1996 [34]	Fine-Wire Iliopsoas	
Hu et al., 2011 [27]	Fine-Wire Iliopsoas Bandpass Filter Between 20 Hz and 1 kHz	
Andersson et al., 1995 [32]	Fine-Wire Psoas Major and Iliacus	
Philippon et al., 2011 [33]	Fine-Wire Iliopsoas	
Jiroumaru et al., 2014 [28]		Active electrode iliacus

Note. Hz, hertz; kHz, kilo-hertz.

## Appendix E

**Table A6:** Individual Exercise Activation Levels of Iliacus in %MVIC.

Exercise	Average %MVIC Activation of Iliacus
Bilateral leg lift	86
Hip flexion bent supported legs	80
Unilateral leg lift	68
Straight leg raise isometric @60° with 20° ER and 30° ABD	65.2
Hip flexion straight supported legs	60
Straight leg raise isometric @60° with 30° ABD	59
Straight leg raise isometric @45° with 30° ABD	48.9
Straight leg raise isometric @60° with 20° ER	47.6
Straight leg raise isometric @45° with 20° ER and 30° ABD	46.7
Straight leg raise isometric @60°	44.1
Straight leg raise isometric @45° with 20° ER	41.2
Straight leg raise isometric @45°	40.6
Straight leg raise isometric @30°	38.4
Straight leg raise isometric @30° with 20° ER and 30° ABD	36.7
Straight leg raise isometric @30° with 20° ER	35
Straight leg raise isometric @30° with 30° ABD	32.5
Incomplete bent unsupported leg task	29
Trunk flexion bent legs	16.5

Note. ABD, abduction; ER, external rotation; MVIC, maximum volitional isometric contraction.

**Table A7:** Individual Exercise Activation Levels of Psoas Major in %MVIC.

Exercise	Average %MVIC of Psoas Major
Straight leg raise isometric @60° with 20° ER and 30° ABD	67.1
Straight leg raise isometric @60°	60.8
Straight leg raise isometric @60° with 20° ER	60.8
Straight leg raise isometric @60° with 30° ABD	54.3
Straight leg raise isometric @45° with 20° ER and 30° ABD	50.3
Straight leg raise isometric @45°	48.8
Straight leg raise isometric @45° with 30° ABD	45.6
Straight leg raise isometric @30° with 20° ER	44.5
Straight leg raise isometric @30° with 20° ER and 30° ABD	41.1
Straight leg raise isometric @45° with 20° ER	37.7
Straight leg raise isometric @30° with 30° ABD	35.9
Hold at top straight leg raise	35
Straight leg raise isometric @30°	34
Late concentric straight leg raise	30
Mid concentric straight leg raise	15
Mid eccentric straight leg raise	15
Early concentric straight leg raise	10
Late eccentric straight leg raise	10

Note. ABD, abduction; ER, external rotation; MVIC, maximum volitional isometric contraction.

**Table A8:** Individual Exercise Activation Levels of Iliopsoas in %MVIC.

Exercise	Average %MVIC Iliopsoas
Eccentric leg raise	23.6
Straight leg raise	17.6
Eccentric sit up	15.8
Sit up	13.2

Note. ABD, abduction; ER, external rotation; MVIC, maximum volitional isometric contraction.

**Table A9:** Individual Exercise Activation Levels of Iliacus in Amplitude.

Exercise	Average EMG Amplitude (µV) of Iliacus
One leg standing and other flexed at the hip ipsilateral @90°	99
One leg standing and other flexed at the hip ipsilateral@60°	75
Static leg lift at 60° ipsilateral	59
Maximal straight leg abduction ipsilateral	56
One leg standing one leg flexed to 90 at the hip and knee ipsilateral	55
Static leg lift at 60° bilateral	55
ASLR ipsilateral with weight	50
One leg standing and other flexed at the hip ipsilateral @30°	43
60° static angle with support and straight legs	42
ASLR ipsilateral	40
Extension max contralateral	26
Hyperlordosis seated	22
Extension 30° contralateral	16
Maximal straight leg abduction contralateral	16
Static lateral flexion to the contralateral side	16
Static lateral flexion to the ipsilateral side against gravity	16
Sitting with a straight back	4
One leg standing and other flexed at the hip contralateral @30°	1
ASLR contralateral	0
ASLR contralateral with weight	0
Standing	0
Standing with trunk flexed 30° at the hip	0
One leg standing and other flexed at the hip ipsilateral @0°	0
One leg standing and other flexed at the hip contralateral @0°	0
One leg standing and other flexed at the hip contralateral @60°	0
One leg standing and other flexed at the hip contralateral @90°	0
One leg standing one leg flexed to 90 at the hip and knee contralateral	0
Extension 30° ipsilateral	0
Extension max ipsilateral	0
Static lateral flexion to the ipsilateral side	0

Note. ASLR, active straight leg raise; EMG, electromyography.

**Table A10:** Individual Exercise Activation Levels of Psoas Major in Amplitude.

Exercise	Average EMG Amplitude (µV) of Psoas Major
One leg standing and other flexed at the hip ipsilateral @90°	85
Static leg lift at 60° bilateral	59
Static leg lift at 60° ipsilateral	58
Static lateral flexion to the ipsilateral side against gravity	54
One leg standing and other flexed at the hip ipsilateral @60°	52
60° static angle with support and straight legs	52
Maximal straight leg abduction ipsilateral	36
One leg standing one leg flexed to 90 at the hip and knee ipsilateral	34
One leg standing and other flexed at the hip ipsilateral @30°	21
Hyperlordosis seated	17
Static lateral flexion to the contralateral side	16
ASLR contralateral with weight	10
ASLR ipsilateral with weight	10
Sitting with a straight back	9
ASLR contralateral	6
ASLR ipsilateral	6
Extension 30° contralateral	4
Extension max contralateral	4
Maximal straight leg abduction contralateral	4
One leg standing and other flexed at the hip contralateral @90°	3
Extension max ipsilateral	2
One leg standing and other flexed at the hip contralateral @30°	1
One leg standing and other flexed at the hip contralateral @60°	1
Standing	0
Standing with trunk flexed 30° at the hip	0
One leg standing and other flexed at the hip ipsilateral @0°	0
One leg standing and other flexed at the hip contralateral @0°	0
One leg standing one leg flexed to 90 at the hip and knee contralateral	0
Extension 30° ipsilateral	0
Static lateral flexion to the ipsilateral side	0

Note. ASLR, active straight leg raise; EMG, electromyography.

**Table A11:** Individual Exercise Activation Levels of Iliopsoas in Amplitude.

Exercise	Average EMG Amplitude (µV) of Iliopsoas
60% MVC with immersion	514
40% MVC with immersion	334
60% MVC without immersion	252
40% MVC without immersion	215
20% MVC with immersion	209
20% MVC without immersion	185
Supine hip flexion concentric	17.5
Sidelying hip abduction-ER concentric	16
Supine hip flexion eccentric	14.6
Traditional hip clam concentric	11.9
Sidelying hip abduction-ER eccentric	11.1
Traditional hip clam eccentric	8
Sidelying hip abduction-wall concentric	7.8
Hip clam-neutral concentric	7.8
Sidelying hip abduction-wall eccentric	6.3
Hip clam-neutral eccentric	4.8
Resisted hip extension concentric	4.4
Resisted knee flexion concentric	4.1
Resisted hip extension eccentric	3.9
Resisted knee extension concentric	3.8
Resisted knee flexion eccentric	3.7
Sidelying hip abduction concentric	3.6
Stool hip rotations eccentric	3.5
Resisted knee extension concentric	3.5
Stool hip rotations concentric	3.4
Sidelying hip abduction eccentric	3.3
Double-leg bridge concentric	3
Double-leg bridge eccentric	2.7
Single leg bridge concentric	2.5
Prone heel squeeze concentric	2.3
Single leg bridge eccentric	2.1

Note. ASLR, active straight leg raise; EMG, electromyography; ER, external rotation; MVC, maximum volitional contraction.

**Table A12:** Individual Exercise Activation Levels of Iliopsoas in RMS.

Exercise	RMS Value of Iliopsoas
Hip flexion angle @30°	1.1
Hip flexion angle @60°	1.05
Hip flexion angle @0°	1
Hip flexion angle @-10°	0.9

Note. RMS, root mean square.
